# Incidental extramammary findings on breast magnetic resonance imaging: Prevalence and recommended diagnostic follow-up

**DOI:** 10.1371/journal.pone.0319373

**Published:** 2025-12-03

**Authors:** Jaber Hussain Alsalah

**Affiliations:** 1 Department of Radiological Sciences, Faculty of Applied Medical Sciences, King Abdulaziz University, Jeddah, Saudi Arabia; 2 King Fahd Medical Research Center, King Abdulaziz University, Jeddah, Saudi Arabia; 3 Smart Medical Imaging Research Group, King Abdulaziz University, Jeddah, Saudi Arabia; Link Campus University, ITALY

## Abstract

Among the current clinical imaging modalities, magnetic resonance imaging (MRI) has the highest sensitivity for breast cancer detection. Only a few studies have been conducted on the extramammary findings of breast MRI; nonetheless, incidental extramammary findings on the breast MR images of the patients are likely to be malignant and may influence staging and treatment. The aim of this study was to assess the prevalence of extramammary findings on breast MRIs and explore the appropriate diagnostic steps. This retrospective study included 234 patients (mean age: 55.8 years) who underwent breast MRI scans at King Abdul-Aziz University Hospital between 2013 and 2020. Reports were reviewed to identify incidental findings, categorized as pathological findings or congenital anomalies. The included patients had confirmed malignant tumors and no prior breast MRI findings. The patient demographics, MRI findings, and indications were recorded. Nineteen incidental extramammary findings were recorded in 16 of the 234 patients with breast cancer whose records were reviewed. The most common sites were the musculoskeletal system (21%), liver (37%), and lungs (21%). Pleural and chest wall involvement was found in two instances (10.5%). One patient (6%) underwent a biopsy, while 15 patients (94%) underwent further imaging. Incidental extra-mammary findings on breast MRI should not be neglected. This study showed that breast MRI examinations were associated with a 7% rate of extramammary findings, suggesting the need for careful evaluation during routine breast MRI. These findings may require further diagnostic evaluation depending on the clinical context. A consistent and systematic diagnostic approach is needed to identify extramammary abnormalities on breast MR scans.

## Introduction

Breast cancer is the most common cancer diagnosed in women and the second most common cause of death from cancer among women worldwide [1–3]. Magnetic resonance imaging (MRI) is a non-invasive imaging modality used to detect, diagnose, and stage breast cancers [4,5]. Breast MRI provides superior soft-tissue contrast and spatial resolution, making it an important tool for evaluating breast cancer recurrence and response to therapies. In addition, its role in clinical practice is expanding [5,6]. MRI is also used to screen females at high risk and examine patients after breast surgery, such as augmentation or reconstruction [7].

The evaluation of breast MRI primarily includes the breast tissue, chest wall, skin, and axillary lymph nodes (LNs); however, the field of view (FOV) of the examination includes other anatomical structures, such as the neck, lung, mediastinum, spine, ribs, sternum, and upper abdomen [8]. These structures are the potential sites of metastasis. Dietzel et al. showed that extending the FOV to the hyoid bone and upper pelvis improves the diagnostic accuracy in detecting distant metastasis [9].

The detection of incidental extra-mammary findings on breast MRI scans may have clinical relevance. The clinical management of incidental extramammary findings largely depends on the patient’s overall clinical context, including cancer stage and history [10]. In patients with known breast cancer, especially those with advanced prognostic stages, incidental findings may represent metastatic disease [10,11]. In contrast, for those without a current breast cancer diagnosis, most incidental findings are benign, and extensive follow-up may not be necessary [10,11].

Breast MRIs are primarily conducted to evaluate the breast tissue; however, the inclusion of adjacent anatomical structures in the FOV often leads to other findings [10]. Extramammary findings on breast MRIs have received little attention; only descriptive research has been conducted [12,13]. The aim of this study was to assess the frequency and location of extramammary findings on breast MRI and explore appropriate diagnostic steps.

## Materials and methods

### Study design

This study was approved by the Institutional Review Board of the King Abdul-Aziz University Hospital (KAUH) (Reference No 426−22) in Jeddah, Saudi Arabia. We retrospectively reviewed the images and reports of patients who underwent breast MRIs at the hospital between 2013 and November 2020. The data for this retrospective study were accessed on October 1, 2022, with the access period spanning from October 2022 to January 2024.

All consecutive patients who underwent breast MRI during the study period were included. Incidental extramammary findings were identified through systematic image review.

### Participants

Overall, 233 breast MRI scans were reviewed. Nineteen incidental extra-mammary findings were identified in 16 patients. The patients’ ages ranged from 28–92 years, with an average age of 59. All breast MRI examination reports and recorded images were reviewed. Variables included in this study were patient age, clinical indications, and MRI findings.

The requirement for written informed consent was waived by the Institutional Review Board owing to the retrospective nature of the study and use of anonymized data.

### Breast MR imaging protocol

Breast MRI was performed using a 3.0 Tesla (3T) Siemens MRI machine (Siemens Healthineers, Munich, Germany). The procedure comprised different MRI sequences: a T2-weighted spectral selection attenuated inversion recovery sagittal sequence (TR 3805 ms, TE 70 ms, voxel 0.5 × 0.5 × 3 [slice thickness]), diffusion-weighted images with a b-value of 1000 (voxel 2.5 × 2.5 × 5 [slice thickness]), T1-weighted turbo spin echo axial sequence (voxel 0.94 × 1.05 × 5 [slice thickness]), performed before and five times after intravenous administration of gadovist (7.5 mL) or uniray prefilled (15 mL), or enhanced T1 high-resolution isotropic volume excitation 6 dynamic sequence (TE 2.5, TR 5.0). The contrast material was injected with a 6-s delay into the dorsal metacarpal vein using an 18-G or 20-G needle at a flow rate of 1.5 mL/s, followed by a flush with 25 mL saline solution. The locations of incidental extramammary findings on breast MRI were categorized into seven organs (liver, lung, bone, mediastinum, pleural or chest wall, supraclavicular LN, or other unknown locations).

### Statistical analysis

Data were reviewed and summarized using frequencies and percentages to represent incidental findings and patient characteristics, respectively. Descriptive statistics, such as means and ranges, were used to assess patient demographics. Incidental findings based on location, tumor characteristics, and diagnostic follow-up methods were presented as absolute frequencies and percentages.

## Results

In this study, breast MRI scans of 233 female patients with breast cancer (age range, 28–92 years; mean age 55.8 years) were reviewed. Nineteen incidental extramammary findings were identified in 16 patients, representing 7% of the total study population. The most common sites were the lungs (n = 4, 21%) and the liver (n = 7, 37%). Additional sites of extramammary findings included the musculoskeletal system (n = 4, 21%), pleura, chest wall (n = 2, 10.5%), and others (n = 10, 5%; Table 1).

**Table 1:** Incidence of extramammary findings based on location.

Location	Incidence	Percentage of all extramammary findings (%)
Liver	7	37
Lung	4	21
Bone	4	21
Pleura and chest wall	2	10.5
Other	2	10.5
Total	19	100

Percentages represent the proportion of total incidental extramammary findings (N = 19).

**Fig 1:**
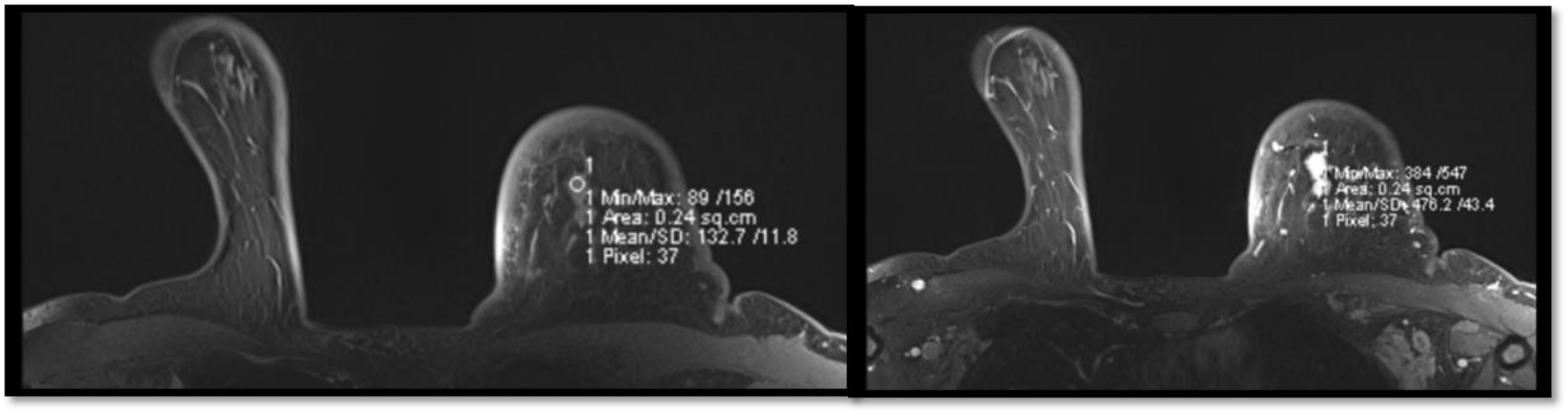
Axial T1-weighted Breast magnetic resonance imaging scans for a 41-year-old patient.

Fig 1 shows MRI images of a 41-year-old female with abnormal enlargement of the left axillary lymph nodes, the largest measuring 1.3 cm. These findings correlate with the mammographic and sonographic results. Multiple hypointense lesions were identified in the liver, thoracic spine, and humeral heads, indicating a high probability of metastasis at these sites. Further evaluation with a bone scan and computed tomography (CT) of the abdomen and pelvis was recommended to confirm the extent of the metastatic disease.

**Fig 2:**
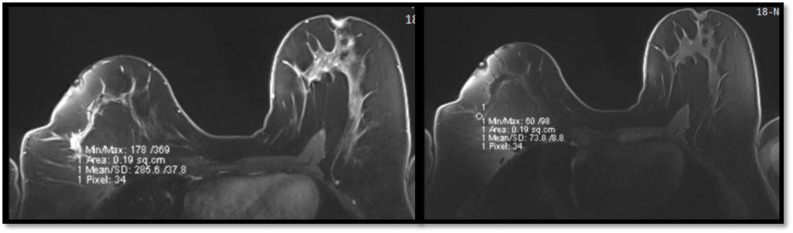
Breast magnetic resonance imaging scans for a 49-year-old patient with an incidental finding.

Fig 2 shows the breast MRI images of a 49-year-old female with an incidental finding of a heterogeneous appearance of the liver parenchyma. There were at least three adjacent malignant-appearing right breast masses with nipple retraction and architectural distortions. An ultrasound-guided core biopsy of the largest breast mass was recommended for definitive pathology.

**Fig 3:**
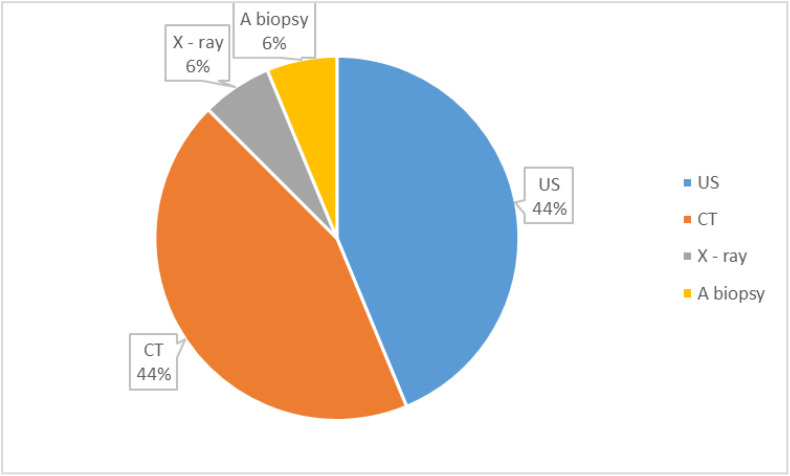
Additional diagnostic examinations for detected extramammary findings.

All patients with extramammary findings were referred for further diagnostic evaluation using either additional imaging techniques or biopsy. Among these patients, 94% (15 16) underwent further radiographic imaging, with ultrasonography (US) and CT being the most common modalities (Fig 3). US or CT was employed in 44% of the cases, highlighting their significant role in diagnostic follow-up. In contrast, X-ray imaging was used in 6% of the cases. Additionally, one patient (6%) required a biopsy for further evaluation of incidental findings. These results underscore the heavy reliance on advanced imaging methods such as US and CT for characterizing extramammary findings, with biopsy reserved for more specific cases where imaging alone is insufficient.

The clinicopathological characteristics of patients with breast cancer with incidental findings are summarized in Table 2. Among the patients, the most common histological type was ductal carcinoma, accounting for 75% of the cases, followed by lobular and invasive mammary carcinomas (each comprising 12.5%). No cases of *in situ* carcinoma were reported. Most tumors were between 1–2 cm (70%); 18.5% were smaller than 1 cm, and 12.5% exceeded 2 cm. Tumor grade analysis revealed that grade 2 tumors were most prevalent (50%), followed by grade 1 (37.5%), with grade 3 representing the smallest proportion (12.5%). These findings indicated the predominance of ductal carcinoma and intermediate-grade tumors in this cohort. The Breast Imaging Reporting and Data System (BI-RADS) scores for patients with incidental findings were higher than those without, ranging from 4–6. This finding suggests that patients with suspected breast lesions may also have a higher likelihood of malignancy at the extramammary sites.

**Table 2:** Clinicopathologic characteristics of patients with breast cancer who had incidental findings.

Variable	Frequency (#)	Percentage (%)
Histological type:		
In situ carcinoma	0	0
Ductal carcinoma	12	75
Lobular carcinoma	2	12.5
Invasive mammary carcinoma	2	12.5
Tumor size:		
< 1 cm	3	18.5
1–2 cm	11	70
> 2 cm	2	12.5
Tumor grade:		
1	6	37.5
2	8	50
3	2	12.5

## Discussion

In this study, the prevalence and clinical significance of incidental extramammary findings on breast MRI were investigated. The findings have profound implications for diagnosis and patient management. The proportion of patients with at least one extramammary finding was 7% in this study, which was similar to previously reported rates on incidental findings in breast MRI examinations. The presence of incidental extramammary findings highlights the importance of routine evaluation of structures beyond the breast parenchyma when radiologists interpret breast MRI scans. Gao et al. emphasized the importance of systematically reviewing the entire FOV during breast MRI interpretation because relevant abnormalities may arise in the chest wall, lungs, liver, or osseous structures, which are not the primary focus of the examination [8].

Consistent with earlier research [8], our study revealed that the liver and lungs were the most common locations of incidental findings during breast MRI examinations; the liver accounted for 37% of extramammary findings in our analysis, followed by the lungs (21%) and the musculoskeletal system (21%). Similarly, Alduk et al. reported that incidental extramammary findings were detected in approximately one-fifth of breast MRI examinations, with the liver and lungs being the most frequent sites, and malignant findings occurring almost exclusively in patients with known breast cancer [12]. This distribution shows that including these anatomical areas in the FOV of breast MRI contributes to incidental detection and warrants further scrutiny, particularly in patients with advanced-stage breast cancer, as these findings may represent metastatic disease [9].

The literature indicates similar trends in the frequency and location of incidental findings [5]. Yang et al. found that liver and lung abnormalities were the most common extramammary findings on breast MRIs, with a prevalence of approximately 5% [10]. The detection of such findings can significantly alter the clinical management of patients, requiring additional imaging and invasive procedures such as biopsies. In our cohort, 94% of patients with incidental findings underwent further diagnostic imaging, and 6% required a biopsy. This finding underscores the importance of a thorough evaluation when extramammary abnormalities are detected, particularly in patients with a known cancer history [5]. Research has shown that early detection can help determine secondary malignancies or other significant pathologies that might not be otherwise diagnosed [5].

The clinical impact of these findings has been emphasized in several studies [10,12]. Alduk et al. also found that most incidental extramammary findings were benign, whereas malignant lesions occurred almost exclusively in patients with a known breast cancer history, highlighting the importance of careful assessment in this population [12]. These findings can change the treatment course in these patients, particularly in cases where the incidental finding is a secondary malignancy. Our findings are consistent with those of prior reports, where additional imaging evaluation was required in several cases; however, the scope of this study did not enable the determination of the impact on treatment decisions [10,12].

Patients with larger tumors may have a higher likelihood of extramammary findings owing to more advanced disease. A higher BI-RADS score was also associated with incidental findings, suggesting a correlation between disease severity and the likelihood of extramammary abnormalities [14–16]. This analysis is supported by the findings of Moschetta et al., who revealed that patients with higher BI-RADS scores were more likely to have incidental findings on breast MRI, many of which could be malignant [12,17].

Despite their potential significance, incidental findings on breast MRIs present clinical challenges. Not all incidental findings are malignant, and the decision to pursue further imaging or biopsy should be balanced against the risk of unnecessary interventions. Gluskin et al. discussed the difficulty in managing incidental findings, highlighting that many are benign and do not warrant aggressive follow-up [17]. However, failure to act on these findings can lead to missed diagnoses, especially in patients with a history of cancer at a high risk of metastasis. Suh et al. emphasized that incidental extramammary findings vary considerably in clinical significance, from benign anatomical variants to metastatic disease, highlighting the need for a systematic and consistent approach for classification and follow-up evaluations [18].

Several researchers have proposed risk-stratified follow-up models that may help standardize management and reduce variability between institutions [15,18]. Current practices vary widely, with some institutions recommending follow-up imaging only for suspicious findings, whereas others pursue further investigation of all incidental findings. The development of these guidelines would help reduce unnecessary interventions and optimize the use of healthcare resources [15,18].

The present study provides valuable insights into the prevalence and clinical significance of extramammary findings on breast MRI; nevertheless, it also highlights the need for further research in this area. Prospective studies with larger cohorts and pathological confirmation of the incidental findings are necessary to better understand the clinical relevance of these findings. In addition, research on the development of risk stratification models to aid clinical decisions regarding incidental findings would be beneficial [15].

Suh et al., among others, have suggested that incidental findings on breast MRI scans should be categorized based on the likelihood of malignancy, with follow-up imaging reserved for findings with suspicious features [15]. This approach could help radiologists better manage incidental findings and reduce the number of unnecessary biopsies, while ensuring that potentially malignant findings are further investigated.

The study has some limitations, including its retrospective, single-center design, lack of histopathological validation for all findings, and the absence of long-term follow-up. Prospective studies are needed to determine how incidental findings influence patient outcomes and to aid in guideline development.

## Conclusion

In conclusion, incidental extramammary findings on breast MRI scans should not be neglected. Our findings emphasized the clinical significance of these extramammary findings on breast MRI scans, particularly in identifying potential malignancies. These findings underscore the importance of careful assessment of structures beyond the breast, as some incidental abnormalities may suggest metastatic involvement and, therefore, require appropriate diagnostic follow-up. However, confirmation using additional imaging and clinical correlations is required in most cases.

## Supporting information

S1 File Supporting Information_re-submitted. (XLSX)
